# Screening of pediatric patients with congenital anomalies of the kidney and urinary tract for associated congenital heart disease in a tertiary care setting

**DOI:** 10.1007/s00467-026-07454-9

**Published:** 2026-07-11

**Authors:** Sahar S. Sheta, Rana Hendawy, Esraa Fathy, Shaimaa Sayed

**Affiliations:** 1 Pediatric Department, Faculty of Medicine, Cairo University, Giza, Egypt; 2 Pediatric Department, Matarya Teaching Hospital, Cairo, Egypt

**Keywords:** Congenital anomalies of the kidney and urinary tract, Congenital heart disease, Vesicoureteral reflux, Pediatrics, Chronic kidney disease

## Abstract

**Background:**

Congenital anomalies of the kidney and urinary tract (CAKUT) are among the most common birth defects and represent a leading cause of chronic kidney disease (CKD) in children. Congenital heart disease (CHD) is the most frequent severe congenital malformation worldwide. Given the shared mesodermal embryologic origin of both the renal and cardiovascular systems, an association between CAKUT and CHD has been proposed. We aimed to screen pediatric patients diagnosed with CAKUT for the presence of associated CHD.

**Methods:**

This cross-sectional analytical study included 300 pediatric patients with CAKUT. Demographic data, type of CAKUT and kidney function status were collected. All patients underwent cardiac evaluation and echocardiography to detect structural heart defects and assess cardiac function. CAKUT types and their association with CHD were analyzed.

**Results:**

The mean age of the cohort was 6.42 years, with a male predominance (68.7%). CHD was detected in 7% of patients, with atrial septal defect (33%) and ventricular septal defect (23%) being the most frequent lesions. 28.5% of these patients with CHD needed surgical intervention. The most common CAKUT subtype was vesicoureteral reflux (27.7%), and it was the most common CAKUT subtype associated with CHD. Fractional shortening impairment was strongly associated with advanced CKD.

**Conclusions:**

The coexistence of CHD among children with CAKUT could emphasize the need for cardiac evaluation and consider the echocardiographic screening in this population. Moreover, the observed link between advanced CKD stage and cardiac dysfunction supports the necessity of integrated nephro-cardiac care in these patients.

**Graphical abstract:**

**Figure:**
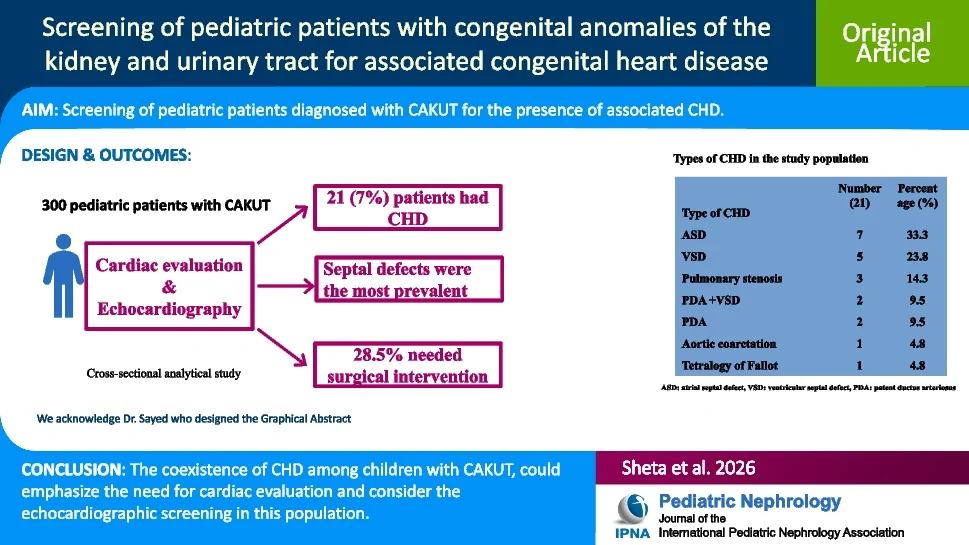
A higher resolution version of the Graphical abstract is available as Supplementary information

**Supplementary Information:**

The online version contains supplementary material available at https://doi.org/10.1007/s00467-026-07454-9.

## Introduction

Congenital anomalies of the kidney and urinary tract (CAKUT) constitute one of the most frequent birth defects and represent the most common cause of chronic kidney disease (CKD) in the first three decades of life [1].

CAKUT include a variety of anatomical and histological malformations implying both obstructive and functional abnormalities. Most patients with CAKUT are diagnosed antenatally, yet many remain undiagnosed until manifestations of symptoms after birth. The complex etiology of CAKUT, along with its multifactorial nature and postnatal disease management, results in progression to CKD [2].

Congenital heart disease (CHD), a wide spectrum of diseases with varied outcomes, is the most common congenital malformation worldwide [3] and the most common severe birth anomaly, affecting almost 1% of infants. Most CHD is genetic, but only 40% of patients have an identifiable genetic risk factor for CHD [4].

Children with CHD have been shown to have a significant incidence of CAKUT [5]. Moreover, children with CAKUT had CHD as the most important co-morbidity [6–8]. Some genetic factors, environmental effects on fetal development, and medications during pregnancy may be the cause of occurrence of CAKUT in CHD [7, 9].

Both the urinary and cardiovascular systems share a mesodermal origin, which means that they may possibly have shared underlying etiologies [10]. In the current study, we aimed to screen pediatric patients diagnosed with CAKUT for the presence of associated CHD in patients who attended a pediatric nephrology and urology outpatient clinic at a tertiary care hospital.

## Methods

This is a cross-sectional prospective analytical study conducted on 300 pediatric patients who were attending the nephrology or urology outpatient clinics of Cairo University children's Hospital from October 2024 to October 2025 and had isolated CAKUT. They were aged from 1 day – 14 years and both sexes were included. We excluded any children with syndromic CAKUT who had any other organ affected except for cardiac manifestations (if present), and those whose parents refused participation in the study.

All patients were subjected to the following:

- *History taking* with special emphasis on demographic data, including: age, gender, family history of CHD and/or CAKUT, antenatal diagnosis for CHD and/or CAKUT, prematurity, whether the patients with CHD were symptomatic or asymptomatic and if they underwent surgical intervention or needed medical treatment.
- *Clinical examination* included cardiac examination. We reviewed each patient’s files for investigations and three blood pressure (BP) measurements at three different visits. Mean values of the BP readings were interpreted using BP percentiles from AAP 2017 BP percentile tables and classified accordingly into: normal BP, elevated BP and hypertension [11].

Results of hemoglobin level, kidney function tests (urea, creatinine), pelvi-abdominal ultrasound and voiding cystourethrogram (if present) were recorded. Estimated glomerular filtration rate (eGFR) was calculated by using the Schwartz formula [12].

Also, echocardiograph was performed for all the patients included during the study to screen for the presence of CHD, its type, and to detect any other abnormalities such as impairment of fractional shortening (FS) and/or left ventricular hypertrophy (LVH). Echocardiograph was performed in a few patients before the study as a part of pre-operative preparation for surgery related to CAKUT. The patients were not known to have CHD except for some patients who needed surgical repair.

### Sample size calculation

Using the statistical program Epi Info version 7.2.4.0., with 5% acceptable margin of error and 95% confidence level we calculated that the estimated minimal sample size was 153 patients. This number could be increased to 184 patients to compensate for possible 20% dropout rate. The sample size was 300 patients.

### Statistical analysis

The statistical analysis of the data was performed using IBM SPSS software version 20.0 (Armonk, NY: IBM Corp, released 2011). Qualitative data were described using number and percent. The Kolmogorov–Smirnov test was used to verify the normality of distribution. Quantitative data were described using range (minimum and maximum), mean, standard deviation, median and interquartile range (IQR). Significance of the obtained results was judged at the 5% level. Chi-square test was used for categorical variables, to compare between different groups. Fisher’s Exact or Monte Carlo correction was used for correction for chi-square when more than 20% of the cells have expected count less than 5. The Student t-test was used for normally distributed quantitative variables, to compare between two studied groups. The Mann–Whitney test was used for abnormally distributed quantitative variables, to compare between two studied groups.

## Results

The median age of the study population was 6.75 years with IQR 8 years. The median duration of follow up was 4 years with IQR 10 years. The demographic profile of the 300 pediatric patients revealed a significant male predominance, with males constituting 68.7% of the study population. The laboratory data showed that 25.7% of the patients had anemia. The mean serum creatinine was elevated with a median of 0.90 mg/dL and IQR 4.5 mg/dL. Patients were staged according to eGFR into the different stages of CKD. The most prevalent stage was CKD stage 5, accounting for 36.0% of the patients (108 cases) (Table 1).

**Table 1:** Demographic data, laboratory findings and CKD stage of the study population

	Number	Percentage (%)
Mean age ± SD (years)	6.42 ± 4.36	
Male	206	68.7
Female	94	31.3
Family history of CAKUT/CHD	9	3.0
Antenatal diagnosis of CAKUT	24	8.0
Prematurity	6	2.0
Mean Hb level ± SD (gram/L)	10.66 ± 1.50	
Normal Hb level	223	74.3
Anemia	77	25.7
Median serum creatinine (IQR) (mg/dl)	0.90 (0.50–5.0)	
CKD stage	**Number**	**Percentage (%)**
Stage 1	63	21.0
Stage 2	53	17.7
Stage 3	45	15.0
Stage 4	31	10.3
Stage 5	108	36.0

*SD* Standard deviation, *CAKUT* Congenital anomalies of the kidney and urinary tract, *CHD* Congenital heart disease, *Hb* Hemoglobin, *IQR* Interquartile range, *CKD* Chronic kidney disease

The types of CAKUT showed a wide spectrum of anomalies, with obstructive uropathies being particularly common. The most prevalent anomalies were vesicoureteral reflux (VUR) in 27.7% of patients followed by posterior urethral valves (PUV) found in 18% of patients. Other significant findings included multicystic dysplastic kidney (MCKD) at 10.4% and hypoplastic kidney at 8.0% (Table 2).

**Table 2:** Different types of CAKUT in the study population

Type of CAKUT	Number	Percentage (%)
Bilateral VUR	48	16
Unilateral VUR	35	11.7
PUV	54	18
Unilateral MCDK	32	10.7
Bilateral hypoplastic kidneys	15	5
Unilateral hypoplastic kidney	9	3
Hypospadias	4	1.3
Bladder exstrophy	9	3
Ectopic ureter	1	0.3
PUJO	17	5.7
Neurogenic bladder	14	4.6
Ureteric stenosis	2	0.7
Horseshoe kidneys	6	2
Hypoplastic bladder	2	0.7
Rt Megaureter	3	1
Unilateral pelvic kidney	8	2.7
Unilateral duplex system	7	2.3
Solitary kidney	34	11.3

*CAKUT* Congenital anomalies of the kidney and urinary tract, *VUR* Vesicoureteral reflux, *PUV* Posterior urethral valve, *MCKD* Multicystic dysplastic kidney, *PUJO* Pelvi-ureteric junction obstruction

Blood pressure readings and echocardiographic parameters of the study population are demonstrated in Table 3. The majority of participants (74.3%) maintained normal BP levels, However, a significant portion showed elevated readings, with 12.3% categorized as having elevated BP and 13.3% diagnosed as hypertensive. 46 patients (15.3%) had LVH and 21 patients (7%) had systolic dysfunction in the form of impaired FS. There was a highly significant association between higher blood pressure and the presence of LVH. So, LVH is strongly associated with hypertension (Table 4).

**Table 3:** Blood pressure readings and echocardiographic parameters of the study population

	Mean ± SD
Systolic blood pressure (mmHg)	100.5 ± 17.73
Diastolic blood pressure (mmHg)	62.93 ± 12.39
IVSD (cm)	0.63 ± 0.18
IVSD Z-Score	1.50 ± 1.59
LVEDD (cm)	3.32 ± 0.74
LVEDD Z-Score	0.18 ± 1.16
LVESD (cm)	2.13 ± 0.50
LVESD Z-Score	0.25 ± 1.01
LVPWD (cm)	0.67 ± 0.41
LVPWD Z-Score	1.25 ± 1.51
EF (%)	63.60 ± 5.47
FS (%)	34.26 ± 4.77

*IVSD* Interventricular septal diameter in diastole, *LVEDD* Left ventricular end-diastolic diameter, *LVESD* Left ventricular end-systolic diameter, *LVPWD* Left ventricular posterior wall diameter in diastole, *EF* Ejection fraction, *FS* Fractional shortening, *SD* Standard deviation

**Table 4:** Association between left ventricular hypertrophy and blood pressure readings

	LVH				Test of Significance	*p* value
	No (*n* = 254)		Yes (*n* = 46)			
	Number	Percentage (%)	Number	Percentage (%)		
Mean systolic blood pressure ± SD (mmHg)	97.17 ± 15.47		118.78 ± 18.44		t = 7.486^*^	< 0.001^*^
Systolic blood pressure percentile						
Normal	209	82.3	14	30.4	χ^2^ = 57.141^*^	< 0.001^*^
Elevated	24	9.4	13	28.3		
Hypertension	21	8.3	19	41.3		
Mean diastolic blood pressure ± SD (mmHg)	61.09 ± 11.21		73.11 ± 13.76		t = 5.597^*^	< 0.001^*^
Diastolic blood pressure percentile						
Normal	185	72.8	20	43.5	χ^2^ = 26.953^*^	< 0.001^*^
Elevated	50	19.7	11	23.9		
Hypertension	19	7.5	15	32.6		

*LVH* Left ventricular hypertrophy, *SD* Standard deviation, *t* Student t-test, χ^2^  Chi square test

There is a statistically significant association between FS impairment and advanced CKD stages (χ^2^ = 13.237, p = 0.005). Patients with FS impairment were predominantly in advanced CKD stage 5 (76.2%). FS impairment is significantly more common among patients with advanced CKD, suggesting that worsening renal function may be associated with deteriorating cardiac systolic performance (Table 5).

**Table 5:** Correlations between fractional shortening impairment and CKD stages

	FS impairment				χ^2^	^Mc^*p* value
	No (*n* = 279)		Yes (*n* = 21)			
	Number	Percentage (%)	Number	Percentage (%)		
CKD stage						
Stage 1	61	21.9	2	9.5	13.237	0.005
Stage 2	51	18.3	2	9.5		
Stage 3	44	15.8	1	4.8		
Stage 4	31	11.1	0	0.0		
Stage 5	92	33.0	16	76.2		

x^2^ Chi square test, *MC* Monte Carlo test, *FS* Fractional shortening, *CKD* Chronic kidney disease

As demonstrated in Table 6, 21 patients (7%) had CHD. Nine of them (42.9%) had cardiac symptoms and 12 patients (57.1%) were asymptomatic and diagnosed by screening, either by cardiological examination and/or echocardiograph. Regarding the cardiac examination; all 7 patients with atrial septal defect (ASD) had normal cardiological examination. Central cyanosis was present since birth in the patient with Tetralogy of Fallot and pan-systolic murmur was present in patients with ventricular septal defect (VSD) who did not undergo corrective surgery. Ejection systolic murmur was present in 3 patients with pulmonary stenosis (PS). Both the patient with patent ductus arteriosus (PDA) and the one with combined VSD and PDA, had corrective surgery. Aortic coarctation was present in 1 patient and it was discovered incidentally by echocardiograph screening.

**Table 6:** Types of CHD and their management in the study population

	Number (21)	Percentage (%)
Type of CHD		
ASD	7	33.3
VSD	5	23.8
Pulmonary stenosis	3	14.3
PDA + VSD	2	9.5
PDA	2	9.5
Aortic coarctation	1	4.8
Tetralogy of Fallot	1	4.8
Cardiac management		
No treatment	12	57.1
Medical treatment	3	14.3
Surgical repair	6	28.6

*CHD* Congenital heart disease, *ASD* Atrial septal defect, *VSD* Ventricular septal defect, *PDA* Patent ductus arteriosus

Also, in Table 7 we show the different types of CHD in relation to types of CAKUT. We attempted to explore if there are specific pairings of heart and kidney defects. Within this dual-diagnosis group, the most common CAKUT anomaly in patients with CHD was VUR (bilateral or unilateral) occurring in 7 of the 21 patients (33.4% combined). It was notably frequent in patients with VSD (5 of the 7 VUR cases were in patients with a VSD or VSD and PDA). Hypoplastic kidney, was found in 5 of the 21 patients (23.8%). This was seen in combination with a variety of heart defects (VSD, ASD, aortic coarctation, pulmonary stenosis). Regarding specific pairings, some potential patterns emerge, although they are based on single or dual cases. For example, both patients with PDA had a right megaureter. The single patient with Tetralogy of Fallot had a pelvic kidney. The patient with aortic coarctation had a hypoplastic kidney (Table 7).

**Table 7:** Types of CHD in relation to type of CAKUT

	CHD															
	Total (*n* = 21)		VSD (*n* = 5)		ASD (*n* = 7)		Aortic coarctation (*n* = 1)		PDA (*n* = 2)		Pulmonary stenosis (*n* = 3)		Tetralogy of fallot (*n* = 1)		PDA + VSD (*n* = 2)	
	No	%	No	%	No	%	No	%	No	%	No	%	No	%	No	%
CAKUT																
Bilateral VUR	3	14.3	2	9.5	1	4.8	0	0.0	0	0.0	0	0.0	0	0.0	0	0.0
Bladder exstrophy	1	4.8	0	0.0	1	4.8	0	0.0	0	0.0	0	0.0	0	0.0	0	0.0
Horseshoe kidney	2	9.5	0	0.0	1	4.8	0	0.0	0	0.0	1	4.8	0	0.0	0	0.0
Hypoplastic kidney	5	23.8	1	4.8	2	9.5	1	4.8	0	0.0	1	4.8	0	0.0	0	0.0
Hypospadias	2	9.5	0	0.0	1	4.8	0	0.0	0	0.0	0	0.0	0	0.0	1	4.8
Lt VUR	3	14.3	1	4.8	1	4.8	0	0.0	0	0.0	0	0.0	0	0.0	1	4.8
Pelvic kidney	1	4.8	0	0.0	0	0.0	0	0.0	0	0.0	0	0.0	1	4.8	0	0.0
PUV	1	4.8	0	0.0	0	0.0	0	0.0	0	0.0	1	4.8	0	0.0	0	0.0
Megaureter	2	9.5	0	0.0	0	0.0	0	0.0	2	9.5	0	0.0	0	0.0	0	0.0
Rt VUR	1	4.8	1	0.0	0	0.0	0	0.0	0	0.0	0	0.0	0	0.0	0	0.0

*VSD* Ventricular septal defect, *ASD* Atrial septal defect, *PDA* Patent ductus arteriosus, *VUR* Vesicoureteral reflux, *PUV* Posterior urethral valve

## Discussion

CAKUT are considered one of the most frequent birth defects and represent the most common cause of CKD in the first three decades of life [1]. Stoll et al. reported that about one third of patients with CAKUT had associated anomalies, including in musculoskeletal, cardiovascular, gastrointestinal and central nervous systems, as well as in the ear, face and neck. They recommended screening of patients with CAKUT for these associated anomalies for proper genetic counselling [13].

CHD has a wide spectrum of disease with varied outcomes and is the most common congenital malformation worldwide [3]. The incidence of CHD in Egyptian children is 1.01/1000 life births [14]. Both the urinary and cardiovascular systems share a mesodermal origin, which means that they may possibly have shared underlying etiologies [10].

This was a cross-sectional analytical study conducted on 300 pediatric patients who had CAKUT. The mean age of the study population was 6.42 years and had a significant male predominance (68.7%). A striking feature is the low rate of antenatal diagnosis (8%), suggesting that many diagnoses were likely made postnatally, possibly following the presentation of symptoms or incidental findings. This may be explained by limited access to advanced prenatal screening and diagnostic facilities, particularly in rural and underserved areas. Additionally, inadequate training of healthcare providers in fetal anomaly detection, inconsistent implementation of routine anomaly scans, and lack of public awareness about the importance of prenatal screening, may have contributed. Moreover, socioeconomic challenges and the high expense of specialized imaging techniques, such as detailed ultrasound and fetal echocardiography, further hinder early and accurate diagnosis [15].

Cardiac anomaly screening should be performed for all pregnancies as part of the routine second-trimester anatomical ultrasound examination [16]. Antenatal diagnosis of CAKUT is also performed during the second trimester [17].

Regarding CAKUT, a prospective study conducted by Atić, in Bosnia and Herzegovina, while showing a similar male-to-female ratio (1.5:1) and a comparable median age of 4.63 years, was conducted on children admitted to a nephrology department or its outpatient clinic, potentially capturing a different severity spectrum [18]. The study by Alp et al., in Turkey presents a much younger cohort, with a median age of just 1.5 months, and a remarkably high rate of prenatal CAKUT diagnosis (60.2%) [19]. Large-scale registry data from Li et al., in China, which included 2,790 newborns, reinforces the male predominance (1.44:1) of CAKUT and highlights the impact of modern healthcare systems with a high prenatal detection rate of 73.87% [20].

In our study, the most common type of CAKUT was VUR at 27.7% followed by PUV at 18%. Other significant findings included multicystic dysplastic kidney (MCDK) (10.4%) and hypoplastic kidney (8%).

Similar to our study, both Atić and Kotb et al. found that VUR (20.8%) was the most frequent malformation [18, 21]. But Li et al. reported hydronephrosis as the most common subgroup, representing 31.79% of all CAKUT [20] and Sayed et al., reported PUV was the commonest abnormality (31.5%) [22].

Regarding prevalence of CHD among patients with CAKUT in our study, CHD was found in 7% (21 out of 300) of the patients. This percentage is remarkably consistent with the 8.89% found in the large newborn cohort by Li et al. [20]. Another Egyptian study conducted on 107 patients with CAKUT for detection of extra-renal manifestations, reported that cardiac features were present in 6.5% but the study did not specify the type of cardiac involvement, either CHD or acquired complications from their renal condition [23].

The study conducted by Alp et al., performed echocardiography only on patients with clinical suspicion (abnormal examination, symptoms, or family history), which constituted 135 out of 806 CAKUT patients. While the prevalence of CHD in this screened subgroup was high (11.2%), they calculated that the prevalence of CHD requiring close follow-up (less than 6 months) in the cohort of 806 patients was 2.2% [19]. This aligns with our study finding that only 6 out of 21 CHD patients (2% of the total study population) required surgical repair. In spite of that, only 7% of study population with CAKUT had associated CHD (21/300), but 28.5% of them (6/21) needed surgical intervention, which is a relatively high percentage. The patients who needed surgical intervention included one patient with Tetralogy of Fallot, 2 patients with VSD, 2 patients with PDA and 1 patient with combined VSD and PDA.

Regarding the types of CHD detected, we found ASD (33%) and VSD (23%) to be the most common. Most of the cases in our study were asymptomatic, with 12 out of 21 CHD accidentally discovered by screening or during preparation for an operation related to CAKUT.

Similar to this present work, the study conducted by Alp et al. presented a notable anomaly, with secundum ASD accounting for an overwhelming 80.2% of the CHD they detected [19]. However, Atić (2016), reported VSD as the most frequent CHD (40.6% of their 32 CHD patients), followed by ASD (15.6%) [18]. This pattern reflects the general epidemiology of CHD in the pediatric population, where septal defects are the most prevalent.

Barakat et al. reported that CHD is the most common anomaly associated with CAKUT, accounting for about 25%. The most prevalent CHD were PDA and VSD, followed by ASD and valvular defects [24].

Regarding specific pairings between CAKUT and CHD subtypes, these could provide clues to shared developmental pathways. In our study, VUR (bilateral or unilateral) was the most common CAKUT, present in one third of these patients, and was frequently associated with VSD. We noted potential pairings, such as PDA with megaureter and Tetralogy of Fallot with a pelvic kidney.

Similar to the present study, Atić found that VUR was the most frequent CAKUT associated with CHD [18], which supports our findings. However, Alp et al., found that isolated hydronephrosis was the most common CAKUT associated with CHD, present in 75.8% of their CHD patients [19].

Both the urinary and cardiovascular systems share a mesodermal origin, which means that they may possibly have shared underlying etiologies [10]. This is based on evidence showing a genetic link between congenital renal malformations and congenital heart malformations [5]. Additionally, pediatricians should be aware of this association and the availability of genetic consultation to avoid delay in diagnosis and therapeutic intervention in order to improve the long-term outcome [24].

In our study, the majority of participants (74.3%) maintained normal BP levels. However, a significant portion showed elevated readings, with 12.3% categorized as having elevated BP and 13.3% diagnosed as hypertensive; almost half of cases were uncontrolled on medications and so developed LVH. A study conducted in Canada by Taha et al., investigating a cohort of 333 children with CAKUT, found the overall hypertension prevalence was 19% (62/333) [25].

In our study, patients with FS impairment were predominantly detected in advanced CKD stage 5 (76.2%), while those without FS impairment were more distributed across the earlier CKD stages. FS impairment is significantly more common among patients with advanced CKD, suggesting that worsening kidney function may be associated with deteriorating cardiac systolic performance. Systolic dysfunction (SD) in patients with CKD is multifactorial, including an increased preload secondary to fluid overload, and LVH which is associated with a reduction in the capillary density, creating disequilibrium between oxygen demand and supply, ultimately culminating in ischemia and myocardial fibrosis, activation of the renin–angiotensin–aldosterone axis and sympathetic nervous system, inflammation, abnormalities of bone and mineral metabolism, and proteinuria all contribute to the pathogenesis of impaired LV systolic myocardial performance in patients with CKD [26].

The study performed by Peter et al., found a 24% prevalence of SD in their cohort of 21 children with CKD; they observed that the occurrence of impaired systolic function was significantly associated with the stage of CKD (p < 0.001), being proportionately most common in stages 3 and 4. The authors note that at stage 5, the initiation of dialysis may improve the systolic performance [26].

In our study, advanced CKD stages were predominantly associated with obstructive uropathy, with VUR representing the largest proportion (49 of 300 patients; 16.3% of the total cohort), followed by PUV (43 of 300 patients; 14.3%). Additionally, MCDK and hypoplastic kidneys contributed notably to CKD progression.

Similarly, VUR is a frequent finding in children with renal hypoplasia/dysplasia. The primary renal damage in these children is usually associated with higher grades of VUR, and both findings may be the result of embryonal abnormality of the ureteral bud. Additionally, the risk of acquired renal scarring is significant in patients with diagnostic delay [27].

In contrast, a cross-sectional study from Germany performed by Isert et al., found that among 407 patients, the most frequent CAKUT lesions were renal hypoplasia/dysplasia (65%), followed by hydronephrosis (43%), and 44% of these patients developed CKD [28].

LVH is a significant form of hypertension-mediated organ damage in children [29]. In our study, 46 out of 300 patients (15.3%) were found to have LVH with strong association with hypertension: systolic hypertension was present in 41.3% of patients with LVH, and diastolic hypertension in 32.6%. Several factors may explain this relationship. First, a proportion of these patients may have had inadequately controlled blood pressure despite being on antihypertensive therapy. Second, even in patients who achieve adequate BP control, regression of LVH is known to occur gradually over time rather than immediately [30].

A large, longitudinal study of 478 children with CKD found that systolic BP was a significant predictor of both left ventricular mass index (LVMI) and the presence of LVH. The study demonstrated that for every 1 standard deviation increase in the SBP z-score, the LVMI increased by between 3.4 to 7.9% [31].

Among the study population, there was a high percentage of anemia. It was reported that CAKUT is commonly associated with anemia [22]. Moreover, anemia is a common morbidity in children with CKD. It is caused by inadequate erythropoiesis, iron deficiency and inflammation [32]. Forty-six percent of the patients included in the current study had advanced CKD (stage 4–5).

## Conclusions

The coexistence of CHD among children with CAKUT, could emphasize the need for cardiac evaluation and consideration of echocardiographic screening in this population. Moreover, the observed link between advanced CKD stage and cardiac dysfunction supports the necessity of integrated nephro-cardiac care in these patients.

To the best of our knowledge, this is the first study to screen pediatric patients diagnosed with CAKUT for the presence of associated CHD in Africa and the Middle East. The current study screened all the patients and was not limited to patients with cardiac symptoms to detect asymptomatic cardiac lesions that may need close follow-up or further cardiac intervention. Additionally, the study included assessment of cardiac function in patients with CAKUT in relation to different CKD stages.

## Limitations

This is a single-center study at a tertiary pediatric hospital without inclusion of a control group. Additionally, the study included a relatively high percentage of patients with advanced CKD stages. Only 7% of the study population with CAKUT had associated CHD (21/300), but 28.5% of them (6/21) needed surgical intervention. So, universal echocardiographic screening cannot be recommended from this study alone and more multi-centric studies on lager scales are required. Additionally, no multivariable analysis was performed.

## Supplementary Information

Below is the link to the electronic supplementary material.

Graphical abstract (PPTX 50 KB)

## Data Availability

All data generated or analysed during this study are included in this published article.
